# The protective effects of Resveratrol against radiation-induced intestinal injury

**DOI:** 10.1186/s12906-017-1915-9

**Published:** 2017-08-16

**Authors:** Heng Zhang, Hao Yan, Xiaoliang Zhou, Huaqing Wang, Yiling Yang, Junling Zhang, Hui Wang

**Affiliations:** 1 Department of Oncology, Institute of Integrative Oncology, Tianjin Union Medical Center, 190, Jieyuan Road, Hongqiao District, Tianjin, 300121 China; 2 Department of Pharmaceutical, Institute of Radiation Medicine, Chinese Academy of Medical Sciences, 238 Baidi Road, Nankai District, Tianjin, China; 3 Department of Breast Cancer Pathology and Research Laboratory, Tianjin Medical University Cancer Institute and Hospital, Huanhuxi Road, Hexi District, Tianjin, China; 4 Tianjin Key Laboratory of Radiation Medicine and Molecular Nuclear Medicine, Chinese Academy of Medical Sciences, Tianjin, China

**Keywords:** Ionizing radiation protection, Small intestine, Mice, Radiation damage

## Abstract

**Background:**

Intestinal injury is a potential cause of death after high-dose radiation exposure. The aim of the present study was to investigate the protective effects of resveratrol against radiation-induced small intestine injury.

**Methods:**

C57BL/6 N mice were irradiated and treated with resveratrol and/or Ex527 (a potent Sirt1 inhibitor), and subsequent examining intestinal morphological changes, and crypt cell apoptosis. Then, the expression and enzyme activity of SOD2 in the small intestine were examined. Furthermore, Sirt1 and acetylated p53 expression was analysed.

**Results:**

Compared to the vehicle control, treatment with resveratrol improved intestinal morphology, decreased apoptosis of crypt cells, maintained cell regeneration, and ameliorated SOD2 expression and activity. Resveratrol also regulated Sirt1 and acetylated p53 expression perturbed by irradiation in the small intestine. The protective effect of resveratrol against ionizing radiation induced small intestine injury was significantly inhibited by Ex527.

**Conclusion:**

Our results suggest that resveratrol decreases the effects of radiation on intestinal injury at least partly via activation of Sirt1.

**Electronic supplementary material:**

The online version of this article (doi:10.1186/s12906-017-1915-9) contains supplementary material, which is available to authorized users.

## Background

Radiation therapy (RT) plays an important role in the treatment of a wide variety of tumors, and in some cases, it has been proven the single best therapeutic approach. Although great advances have been achieved in RT, normal tissues surrounding the tumors are still subject to damage by the treatment [1]. For patients with abdominal or pelvic tumors, high-dose RT is limited by the toxicity of the radiation on the small intestine. Ionizing irradiation (IR)-mediated toxicity is largely defined as clonogenic cell death and apoptosis in the crypt cells, resulting in insufficient replacement of villus epithelium, breakdown of the mucosal barrier leading to mucositis, and prominent inhibition in the compensatory proliferative reactions [2, 3]. The toxic effects of RT on patients include anorexia, vomiting, diarrhea, dehydration, systemic infection, and in extreme cases, septic shock and death [4, 5]. Such side effects may lead to a reduced quality of life for the patient and necessitate radiation dose reduction, which limits treatment success.

Resveratrol (3,4′,5-tri-hydroxy-trans-stilbene, Rev) is a natural polyphenol, mostly found in *Polygonum cuspidatum*, grapes, and red wine [6]. Some of its reported pharmacological effects include anti-apoptotic, anti-inflammatory, and anti-aging effects [6–8]. Resveratrol has been shown to be a scavenger of hydroxyl, superoxide, and metal-induced radicals [9], which is associated with its protective effect on normal tissue cells against IR-induced injury [10]. Recent studies have reported that resveratrol is a putative activator of Sirtuin1 (Sirt1) [9], a member of the mammalian sirtuin family [11]. Sirt1 has been found to deacetylate various transcription factors that trigger cell defences and survival in response to stress and DNA damage such as activation of DNA repair system, enhancement of cell cycle progression, and prevention of cellular senescence and apoptosis [12]. Some studies revealed that overexpression of Sirt1 is associated with resistance to radiotherapy and chemotherapy [13–16]. It was also reported that resveratrol ameliorated total body irradiation-induced bone marrow injury [10], mesenchymal stem cells’ inflammation [17], and ovarian inflammation through Sirt1 activation [18].

We recently showed that resveratrol protected hematopoietic cells from IR-induced injury at least partly via Sirt1 activation in mice [10]. We also found that resveratrol treatment effectively inhibited ionization-induced oxidative stress, particularly in bone marrow hematopoietic cells [10]. In this study, we explored the possibility and mechanisms that resveratrol may protect the intestinal tissue from IR-induced injury.

## Methods

### Animals and experimental model

Male C57BL/6 N mice (8–10 weeks of age) were purchased from the Institute of Laboratory Animal Sciences (Peking Union Medical College, Beijing, China). Animals were divided randomly into 4 groups (6 mice per group): (a) control; (b) vehicle + IR; (c) Rev. + IR; and (d) Rev. + Ex527 + IR. They were bred at the certified animal care facility at the Institute of Radiation Medicine of the Chinese Academy of Medical Sciences, where they were maintained in a room at 23 °C ± 2 °C, with a relative humidity of 50% ± 5%, artificial lighting from 08:00–20:00 h, and 13 ~ 18 air changes per hour, and they received food and water ad libitum. All experimental procedures were carried out in accordance with the NIH Guidelines for the Care and Use of Laboratory Animals and were approved by the Institutional Animal Care and Use Committee of Tianjin Union Medical Center.

Resveratrol (R5010, Sigma Biotechnology, St. Louis, MO, USA) was dissolved in 96% ethanol to achieve a stock solution concentration of 50 mg/mL. Before gavage, the stock solution was further diluted in distilled water for a final concentration of 2.5 mg/mL [10]. Individual mice in the *c* and *d* group received a dose of 40 mg/kg resveratrol administered by gavage every day for 1 day before irradiation and then 5 days after irradiation. Ex527 (C_13_H_13_ClN_2_O) (E7034, Sigma Biotechnology, St. Louis, MO, USA), a potent Sirt1 inhibitor, was reconstituted in 1% DMSO and 30% PEG-400 and 1% Tween 80. Individual mice in the *d* group received a dose of 10 mg/kg EX527 administered via intraperitoneal injection every day for 1 day before irradiation and then 5 days after irradiation at the same time as Rev.

Mice of group *b*, *c*, and *d* were shielded for the head, thorax, and extremities, and were kept in perforated plastic cages. A single dose of 7 Gy partial-body irradiation (PBI) was accomplished using an Exposure Instrument Gammacell-40 ^137^Cs irradiator (Atomic Energy of Canada Ltd) at a dose rate of 0.73 Gy/min from a source-surface distance of 75 cm. Mice were irradiated on a rotating platform and mice in the control group were sham-irradiated.

### Histological analysis

At six day after IR, mice were sacrificed and small intestines were harvested and flushed with saline. Two-centimetre–long sections were fixed in 4% neutral buffered formalin for 16 h and embedded in paraffin using standard procedures. Paraffin-embedded sections were cut into 4 μm sections that were subsequently stained with hematoxylin-eosin (H&E) and analysed under a microscope (BX51, Olympus, Japan). Images were captured with a Nikon D90 camera (Nikon, Japan). For morphological analysis, six circular transverse sections were analysed per mice in a blinded fashion to establish the mean value of the length of the ten longest villi, number of crypt cells per villus section, number of crypts per circumstance, and the basal lamina length [19, 20] by using the Image-Pro Plus 5.1 software (IPP, Media Cybernetics, Rockville, MD, USA) [21].

### Immunohistochemical staining

The 4-μm–thick sections from paraffin-embedded small intestine sections were deparaffinized and rehydrated using xylene and ethanol, and immersed in a 3% hydrogen peroxide solution for 10 min to block endogenous peroxidase. The sections were then boiled for 30 min in 10 mM/L citrate buffer solution (pH 6.0) for antigen retrieval. Slides were incubated with 5% skimmed milk for 20 min and then with anti-Ki67 protein antibody (1:2000, ab66155, Abcam Biotechnology, Cambridge, MA, USA) and anti-cleaved caspase-3 antibody (1:400, Clone # 269518, R&D Systems, Minneapolis, MN, USA) at 4 °C for 16 h, and visualized using PV-6001 Polymer Detection System (Golden Bridge International Corp, Wharton, TX, USA) following the manufacturer’s instructions. Subsequently, the slides were incubated with 3,3′-diaminobenzidine tetrahydrochloride-H_2_O_2_ solution for visualization, and counterstained with hematoxylin. For analysis, four views at 400-fold magnification were chosen randomly for each slide. The images were captured and positive staining was quantified objectively by the IPP software as described previously [21] in a blinded fashion.

### Western blot analysis

Thirty milligrams of small intestinal tissue was homogenized and lysed in M-PER mammalian protein extraction reagent (Thermo Scientific, Rockford, IL, USA). Protein concentration was estimated using the bicinchoninic acid protein assay kit (Beyotime Institute of Biotechnology, Jiangsu, China). Cell lysates (50 μg) were loaded onto 5–10% gradient polyacrylamide gels. Proteins were electroblotted onto polyvinylidene difluoride membranes (Millipore, MA, USA) and immunolabeled using anti-SIRT1 antibody (1:800, ab28170, Abcam Biotechnology, Cambridge, MA, USA), anti-acetylated p53 antibody (1:600, ab61241, Abcam Biotechnology, Cambridge, MA, USA), anti-SOD2 antibody (1:1000, ab68155, Abcam Biotechnology, Cambridge, MA, USA), and antibodies against β-actin (1:2000, Santa Cruz Biotechnology, Santa Cruz, CA, USA). Enhanced chemiluminescence plus reagent (Boster Biotechnology Co., Wuhan, China) was used for chemiluminescent signal detection. Quantitative analysis was performed using Quantity One software version 4.6.2 (http://www.bio-rad.com/en-us/product/quantity-one-1-d-analysis-software).

### Analysis of enzymatic activity of SOD2

SOD2 enzymatic activities in small intestinal tissue were analysed using a SOD2 assay kit (Beyotime Institute of Biotechnology, Jiangsu, China), respectively, following the manufacturer’s instructions [10].

### Statistical analysis

Data were examined using SPSS 16.0 software (SPSS, Inc., Chicago, IL, USA). In the event that ANOVA justified post hoc comparisons between group means, Student–Newman–Keuls test was used for multiple comparisons. Differences were considered significant when *p* < 0.05.

## Results

### Clinical changes of mice

At six day after IR, mice in each group had no death. In control group*,* mice had smooth and shiny fur, normal diet and activity, and weight gain (Additional file 1: Figure S1). Mice treated with IR showed withered hair, inappetence, slow in action, and no weight growth. Compared with group of IR, mice treated with Rev. had better skin luster, diet, and activity, and increased body weight. Mice treated with Rev. and EX527 were similar to IR group, and the body weight did not increase.

### Resveratrol improves intestinal cell morphology in irradiated mice

**Fig. 1:**
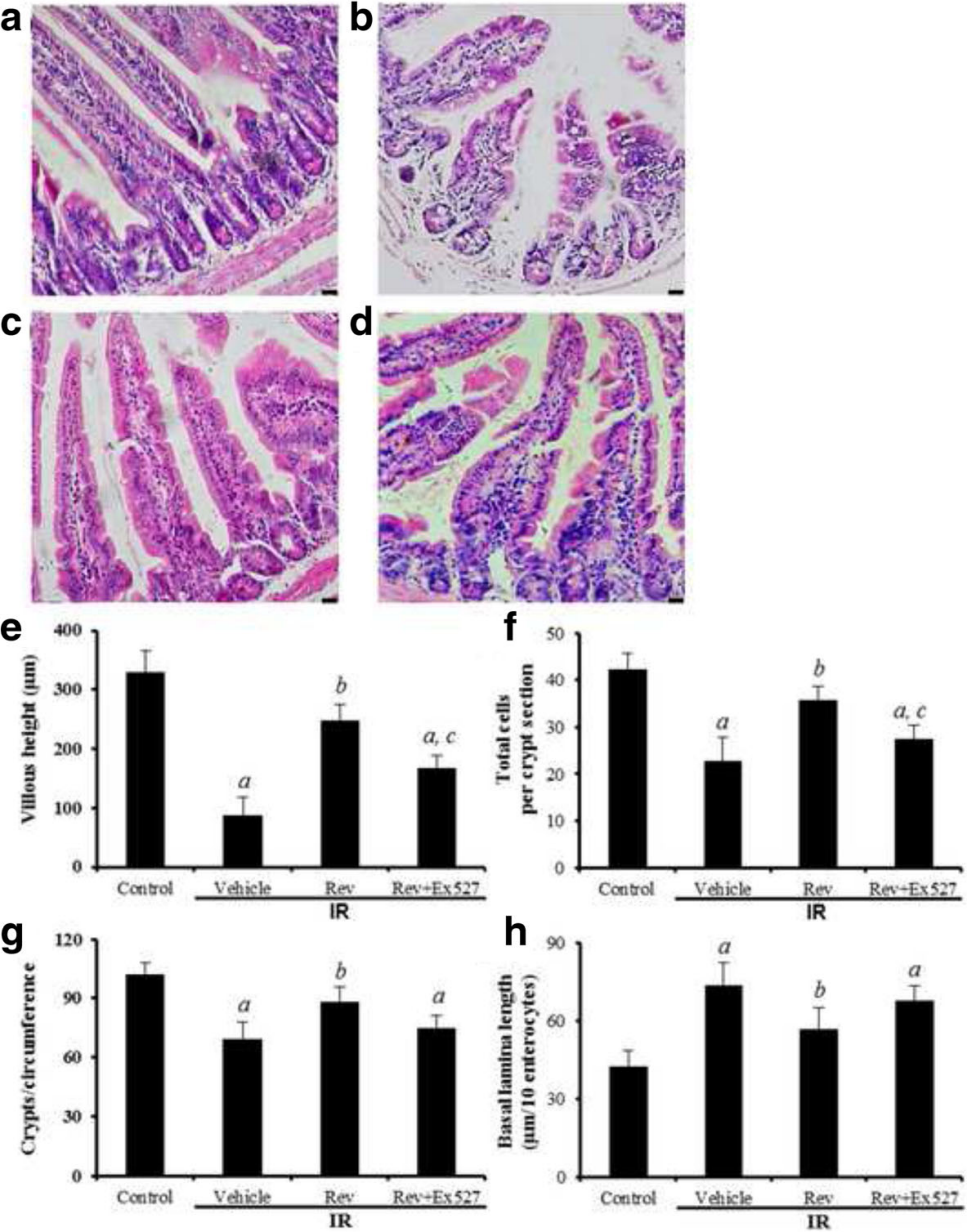
Resveratrol improve intestinal cell morphology in irradiated mice. Representative images showing the villous, crypts, and the basal lamina length in cross-sections of the small intestine stained with H&E: **a** sham control, (**b**) vehicle-treated irradiation mice, (**c**) resveratrol treated irradiation mice, and (**d**) resveratrol plus Ex527 treated irradiation mice. Bar graphs showing Quantitative analysis of the villus height (**e**), the number of the crypt cells (**f**), the number of the crypts (**g**), and the basal lamina length (**h**). The data are presented as means ± SE. *n* = 6 mice per group. ^a^ *p* < 0.01 vs control; ^b^ *p* < 0.05 vs IR; ^c^ *p* < 0.05 vs IR + Rev. Scale bar: 20 μm

The morphological changes observed in mouse jejunum are shown in Fig. 1. Tissue sections of intestine from the control group showed normal morphology. At 6 day after IR, irradiated mice showed significantly lower villus, fewer surviving crypts, and longer basal lamina length (*p* < 0.05). In comparison to irradiated mice exposed to vehicle treatment, resveratrol-treated mice showed more surviving crypts, increased villi length and shorter basal lamina length (*p* < 0.05). These results suggest that resveratrol treatment significantly improved intestinal cell morphology in irradiated mice.

### Resveratrol maintains the regeneration of intestinal cells in irradiated mice

**Fig. 2:**
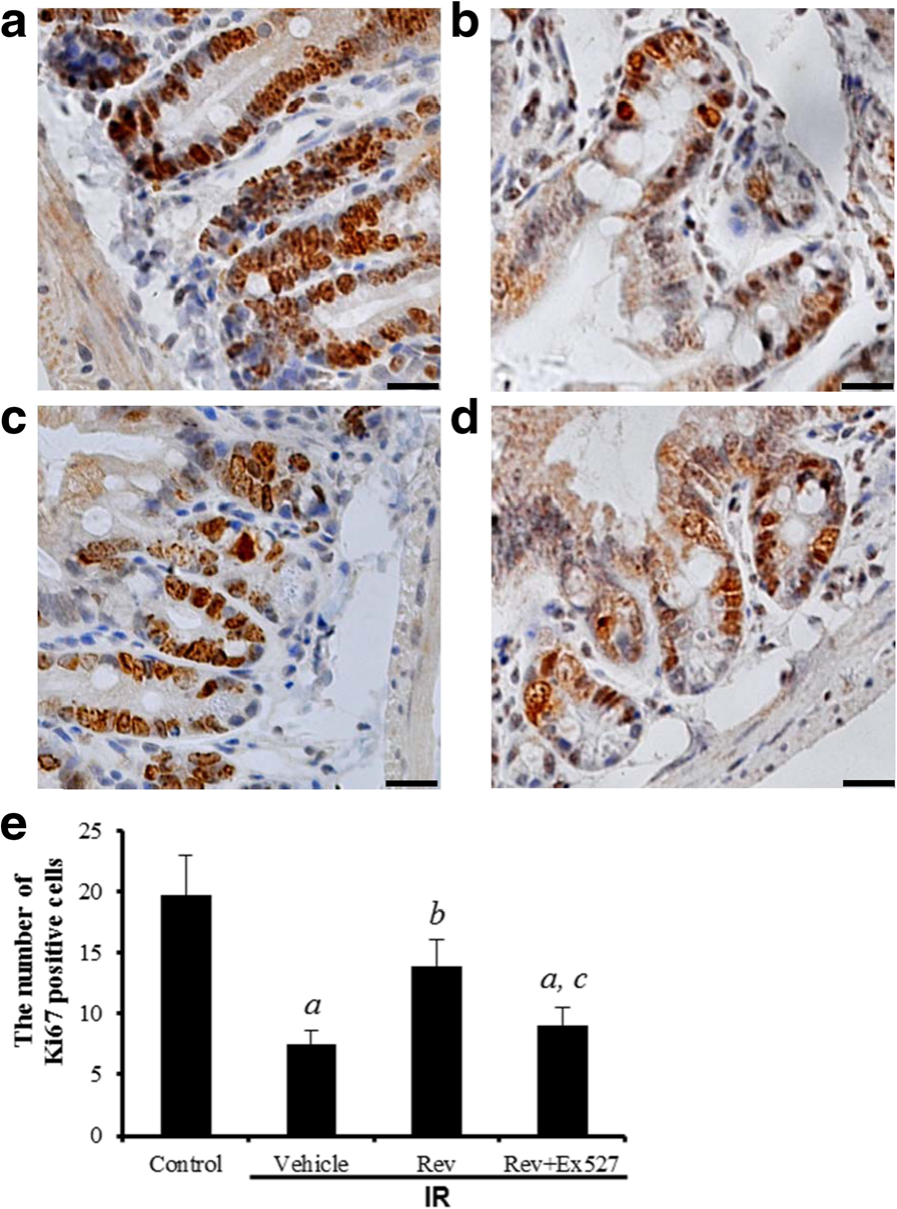
Analysis of IHC of intestinal crypts stained with an antibody against Ki-67. **a** sham control mice, (**b**) vehicle-treated irradiation mice, (**c**) resveratrol treated irradiation mice, and (**d**) resveratrol plus Ex527 treated irradiation mice. (e) Bar graphs showing Quantitative analysis of the Ki-67 expression of crypt cells. The data are presented as means ± SE. *n* = 6 mice per group. ^a^ *p* < 0.01 vs control; ^b^ *p* < 0.05 vs IR; ^c^ *p* < 0.05 vs IR + Rev. Scale bar: 20 μm

Proliferation of crypt cells was identified by immunohistochemical staining of Ki-67 [22] (Fig. 2). Most Ki-67 positive cells were detected in the crypts (Fig. 2a). The number of Ki-67 positive cells decreased in irradiated mice (Fig. 2b, e), corresponding to the decrease in surviving crypts at 6 day after IR. A greater number of Ki-67 positive cells was observed in the resveratrol-treated group than in the IR group (Fig. 2c, e), similar to the increase in villus height and number of crypt cells (Fig. 1). The difference in Ki-67 expression between the IR group and the resveratrol-treated group indicates resveratrol helps maintain regeneration of intestinal crypt cells in mice exposed to radiation.

### Resveratrol reduces apoptosis in the intestinal cells of irradiated mice

**Fig. 3:**
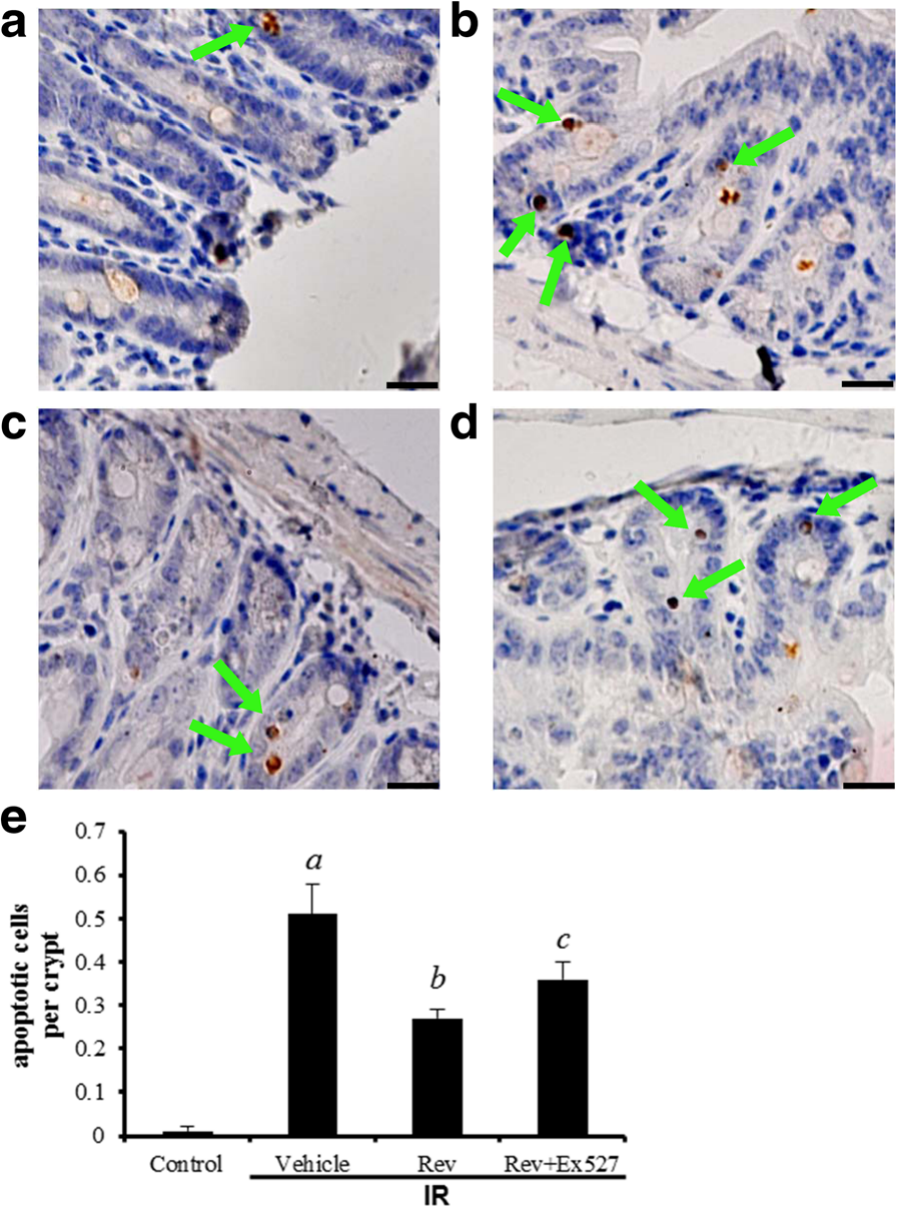
Analysis of intestinal crypt cell apoptosis in irradiated mice. Photomicrograph of cleaved caspase-3 stained section of mouse intestine: (**a**) sham control, (**b**) vehicle-treated irradiation mice, (**c**) resveratrol treated irradiation mice, and (**d**) resveratrol plus Ex527 treated irradiation mice. Arrows indicate positive staining. **e** Bar graphs showing quantitative analysis of the apoptotic cells. The data are presented as means ± SE. *n* = 6 mice per group. ^a^ *p* < 0.01 vs control; ^b^ *p* < 0.05 vs IR; ^c^ *p* < 0.05 vs IR + Rev. Scale bar: 20 μm

Figure 3 shows the apoptotic cells observed in small intestine crypts at 6 day after IR. In the crypts, apoptosis was recognized by cleaved caspase-3 antibody staining after IR (Fig. 3). The stained products strongly resembled the typical morphology of apoptosis when evaluated using optical microscopy. Radiation exposure can increase the number of apoptotic nuclei in the small intestine crypts of mice. As shown in Fig. 3e, at day 6 after IR, the resveratrol-treated mice showed a significantly lower number of apoptotic cells than irradiated mice treated with the vehicle (*p* < 0.01).

### Resveratrol ameliorates IR-induced depression in SOD2 expression and activity

**Fig. 4:**
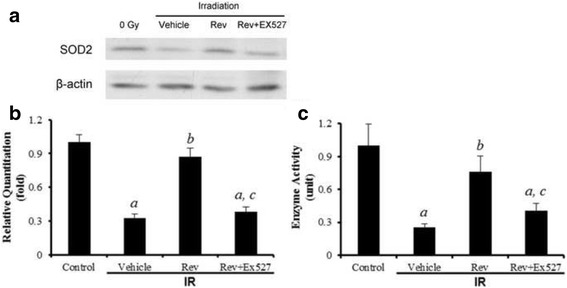
Resveratrol ameliorate IR-Induced reductions in SOD2 expression and activity. **a** The levels of SOD2 expression; (**b**) Quantitative analysis of the SOD2 expression; (**c**) Quantitative analysis of the enzyme activity of SOD2. The data are presented as means ± SE. *n* = 6 mice per group. ^a^ *p* < 0.01 vs control; ^b^ *p* < 0.05 vs IR; ^c^ *p* < 0.05 vs IR + Rev

Increased production of reactive oxygen species (ROS) by irradiated cells has been largely attributed to the dysfunction of mitochondria [23]. SOD2 is a primary oxidative stress defence enzyme in mitochondria, which converts superoxide radical into hydrogen peroxide (H_2_O_2_). It was reported that IR-induced ROS stress contributes to IR-induced bone marrow failure in hematopoietic cells partly via downregulating the activity of SOD2 [10]. In this study, the expression of SOD2 in small intestinal tissue exposed to IR and treated with the compounds was investigated. As indicated in Fig. 4, IR exposure significantly downregulated the expression of SOD2 at 6 day after IR. Following treatment with resveratrol, the expression of SOD2 was upregulated. The modulation of SOD2 expression by IR and resveratrol was also confirmed by performing enzymatic assays (Fig. 4c).

### Resveratrol protects the intestine against radiation-induced injury at least in part via activation of Sirt1

**Fig. 5:**
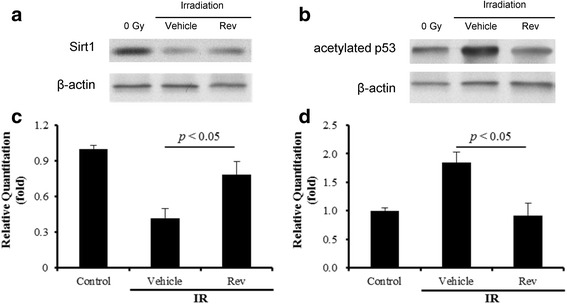
Resveratrol moderates Sirt1, and acetylated p53 expression perturbed by irradiation in mouse small intestine. **a** The levels of Sirt1 expression; **b** the levels of acetylated p53 expression; (**c**) Quantitative analysis of the Sirt1 expression; (d) Quantitative analysis of the acetylated p53 expression. The data are presented as means ± SE. *n* = 6 mice per group

Resveratrol is a putative activator of Sirt1, which is a NAD^+^-dependent deacetylase with many biological functions [11]. Activation of Sirt1 can protect cells from oxidative stress-induced damage in part via deacetylation of several transcriptional factors to regulate the expression of various genes including SOD2 [24]. To gain a better understanding of the mechanisms by which resveratrol protects small intestine from radiation injury, we investigated the effects of resveratrol treatment on Sirt1. As shown in Fig. 5a, IR reduced the expression of Sirt1 in small intestine by 58.1%. This reduction was associated with a significant increase in the acetylation of p53, indicating that IR also decreased Sirt1 deacetylase activity in small intestine (Fig. 5b). These effects of IR on Sirt1 were abrogated by the treatment of resveratrol, suggesting that resveratrol may protect small intestine from IR in part via activation of Sirt1. To test this hypothesis, we first examined whether resveratrol can upregulate the expression and enzyme activity of SOD2 in small intestine in a Sirt1-dependent manner, because SOD2 is one of the most important intracellular antioxidants that can protect cells from radiation injury. As shown in Fig. 4, small intestine of mice treated with resveratrol exhibited a significant increase in SOD2 expression. The increase was inhibited by the addition of Ex527, a potent Sirt1 inhibitor [25]. More importantly, we found that resveratrol could maintain the regeneration and reduce apoptosis of intestinal cells against IR and the protective effect was significantly attenuated by Ex527 (Fig. 1-3). Collectively, these findings suggest that resveratrol protects small intestine from radiation at least in part via activation of Sirt1.

## Discussion

The small intestinal crypt cells are particularly susceptible to IR due to their high rate of proliferation [26]. Recovery from intestinal damage after radiation exposure has been found to depend on clonogenic cell survival in the intestinal crypt [27]. High-dose IR induces loss of proliferative function among intestinal crypt cells and may thus result in increased permeability of the intestinal lining, including permeability to intestinal bacteria, and potentially bacteremia as well as exacerbation of mucosal inflammation [28, 29]. The increased expression of Ki-67, a proliferative marker in the small intestine, in the resveratrol treated group may indicate the recovery of intestinal damage after IR. Thus, resveratrol treatment can also partly improve intestinal morphology after IR-related intestinal injury. These results indicate that resveratrol provides significant protection against intestinal injury following IR.

The mechanisms by which resveratrol regulates the expression of SOD2 in small intestine to protect it from radiation injury have yet to be investigated. Resveratrol is a putative activator of Sirt1 [9]. It has been shown that resveratrol can protects human endothelium from H_2_O_2_-induced oxidative stress and senescence [30], prevents antibody-induced apoptotic death of retinal cells [31] and extend the life span of yeast, worms, *Drosophila melanogaster*, and *Caenorhabditis elegans* [32, 33], in part by activation of Sirt1. Our studies showed that IR reduced the expression and the deacetylase activity of Sirt1 in small intestinal. These effects of IR on Sirt1 were impaired by the treatment with resveratrol, suggesting that resveratrol may protect small intestinal from IR in part via activation of Sirt1. This suggestion is supported by the findings that resveratrol can upregulate the expression of SOD2 in small intestinal and protect small intestinal from IR in a Sirt1-dependent manner. Sirt1 targets histones and non-histones proteins such as FOXO3a, nuclear factor erythroid2-related factor2 (Nrf2), and peroxisome proliferator-activated receptor coactivator1 a (PGC-1a) [11, 24], which may be involved in the regulation of SOD2 expression by resveratrol reported in this study. Recent studies clarified that PGC-1a, a transcriptional coactivator, regulates prooxidant molecules by coordinating mitochondrial biogenesis [34] and the gene expression of antioxidants such as SOD2 [35]. It was reported that increasing PGC-1a levels dramatically protect cultured neural cells from oxidative stressor-mediated death [36]. Furthermore, Nrf2 is a transcription factor that also plays an important role in the regulation of expression of several antioxidant enzymes [37]. Nrf2 knockout animals are sensitive to oxidative stress and radiation-induced tissue injury [38]. Resveratrol is known to disrupt the Nrf2/Keap1 interaction, which can activate Nrf2 to induce the expression of many antioxidants being proposed as protectants against IR [39]. It will be interesting to determine whether these activities of resveratrol play a role in the inhibition of IR induced small intestine injury.

Amifostine (WR-2721) is the only radioprotective drug approved by the FDA for head and neck cancer. It is widely accepted that the radioprotective mechanism of AM occurs via scavenging radiation induced free radicals [40, 41]. Huang et al. showed that this molecule could protect rat intestine up to 18 Gy [42]. Milas et al. also noted that Amifostine could protect the mouse jejunum from IR induced injury [43]. The effective dose and the minimum toxic dose of Amifostine are very close. Due to great individual differences, patients often suffer serious adverse reactions as hypocalcemia, diarrhea, nausea, vomiting, et al. In this study, our date support resveratrol can inhibit IR-induced intestinal injury in a PBI mouse model. Resveratrol may be more reliable than other commonly used radio-protective agents such as Amifostine, particularly considering that resveratrol is a relatively low toxicity and has been widely used as a food supplement for management of various human health conditions. The dose (40 mg/kg·day) of resveratrol used in our study is safely achievable in humans, because the dose of 100 mg/kg·day resveratrol in mice is equivalent to 2 mg/kg·day in humans [44, 45]. Doses of up to 5 g of resveratrol a day have been shown to be safe in a clinical study in humans [46]. However, the protective effect of resveratrol against radiation induced intestine injury is limited, because we found that treatment with resveratrol had no significant effect on that of mice exposed to a higher dose of IR (> 8.0 Gy, data not shown).

In addition, exposure to IR not only induces intestine injury but also causes damage to other tissues such as lung fibrosis and bone marrow suppression. It has been shown that not only cell apoptosis but also chronic oxidative stress is the underlying cause of IR-induced tissue damages. Therefore, it is interesting to examine whether resveratrol may be beneficial for other IR-induced tissue damage.

## Conclusion

The results of this study suggest that administration of resveratrol attenuates radiation induced intestine damage in mice. The study also suggests that resveratrol may protect the intestine from IR-induced injury at least partly via the activation of Sirt1.

## Additional file

Additional file 1: Figure S1. Resveratrol increases body weight in irradiated mice. All animals were weighed 1 h before irradiation and at 6 day after IR and their well-being inspected daily from the initiation of treatment to the end of the study. The data are presented as means ± SE. *n* = 6 mice per group. (DOCX 102 kb)

## Abbreviations

H&E: Hematoxylin-eosin
IR: Ionizing irradiation
Nrf2: Nuclear factor erythroid2-related factor2
PBI: Partial-body irradiation
PGC-1a: Peroxisome proliferator-activated receptor coactivator1 a
Rev: Resveratrol
ROS: Reactive oxygen species
RT: Radiation therapy
