# Supplementary material for: The protective effects of Resveratrol against radiation-induced intestinal injury
Source: BMC Complement Altern Med. 2017 Aug 16;17:410. doi: 10.1186/s12906-017-1915-9 (PMC5559783; doi:10.1186/s12906-017-1915-9)
Supplement: Additional file 1: Figure S1. — Resveratrol increases body weight in irradiated mice. All animals were weighed 1 h before irradiation and at 6 day after IR and their well-being inspected daily from the initiation of treatment to the end of the study. The data are presented as means ± SE. n = 6 mice per group. (DOCX 102 kb) [file 12906_2017_1915_MOESM1_ESM.docx]

Figure 1


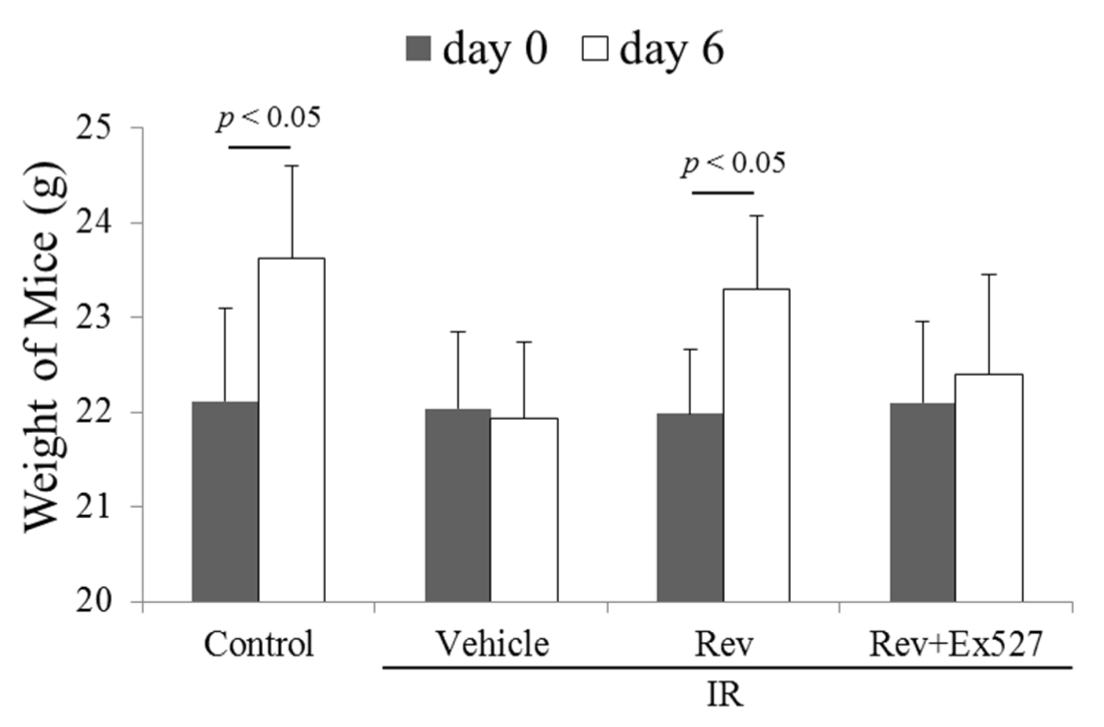


**Figure 1. Resveratrol increases body weight in irradiated mice.** All animals were weighed 1 h before irradiation and at 6 day after IR and their well-being inspected daily from the initiation of treatment to the end of the study. The data are presented as means ± SE. *n* = 6 mice per group.
